# Quantitative Trait Loci Associated with Drought Tolerance in *Brachypodium distachyon*

**DOI:** 10.3389/fpls.2017.00811

**Published:** 2017-05-17

**Authors:** Yiwei Jiang, Xicheng Wang, Xiaoqing Yu, Xiongwei Zhao, Na Luo, Zhongyou Pei, Huifen Liu, David F. Garvin

**Affiliations:** ^1^College of Agronomy, Resources, and Environment, Tianjin Agricultural UniversityTianjin, China; ^2^Department of Agronomy, Purdue UniversityWest Lafayette, IN, United States; ^3^Institute of Horticulture, Jiangsu Academy of Agricultural SciencesNanjing, China; ^4^Department of Agronomy, Iowa State UniversityAmes, IA, United States; ^5^Department of Crop Genetics and Breeding, Sichuan Agricultural UniversityChengdu, China; ^6^College of Life Sciences, South China Agricultural UniversityGuangzhou, China; ^7^Department of Agronomy and Plant Genetics, University of MinnesotaSt. Paul, MN, United States; ^8^Plant Science Research Unit, United States Department of Agriculture, Agricultural Research ServiceSt. Paul, MN, United States

**Keywords:** allelic variation, *Brachypodium distachyon*, drought tolerance, genetic map, QTL mapping, SNP marker

## Abstract

The temperate wild grass *Brachypodium distachyon* (Brachypodium) serves as model system for studying turf and forage grasses. Brachypodium collections show diverse responses to drought stress, but little is known about the genetic mechanisms of drought tolerance of this species. The objective of this study was to identify quantitative trait loci (QTLs) associated with drought tolerance traits in Brachypodium. We assessed leaf fresh weight (LFW), leaf dry weight (LDW), leaf water content (LWC), leaf wilting (WT), and chlorophyll fluorescence (Fv/Fm) under well-watered and drought conditions on a recombinant inbred line (RIL) population from two parents (Bd3-1 and Bd1-1) known to differ in their drought adaptation. A linkage map of the RIL population was constructed using 467 single nucleotide polymorphism (SNP) markers obtained from genotyping-by-sequencing. The Bd3-1/Bd1-1 map spanned 1,618 cM and had an average distance of 3.5 cM between adjacent single nucleotide polymorphisms (SNPs). Twenty-six QTLs were identified in chromosome 1, 2, and 3 in two experiments, with 14 of the QTLs under well-watered conditions and 12 QTLs under drought stress. In Experiment 1, a QTL located on chromosome 2 with a peak at 182 cM appeared to simultaneously control WT, LWC, and Fv/Fm under drought stress, accounting for 11–18.7% of the phenotypic variation. Allelic diversity of candidate genes *DREB2B, MYB*, and *SPK*, which reside in one multi-QTL region, may play a role in the natural variation in whole plant drought tolerance in Brachypodium. Co-localization of QTLs for multiple drought-related traits suggest that the gene(s) involved are important regulators of drought tolerance in Brachypodium.

## Introduction

Numerous morphological, physiological, and biochemical responses are altered in plants exposed to drought stress (Farooq et al., 2009). The adaptive mechanisms of drought tolerance at whole-plant and cellular levels increase plant survival from water deficit conditions. Drought tolerance traits are complex, controlled by multiple genes, thus posing a challenge to fully revealing genetic control of functional physiological traits for drought tolerance across variable environments (El-Soda et al., 2014; Gupta et al., 2017). Nevertheless, detection of quantitative trait loci (QTLs) for controlling whole-plant physiological responses to drought stress provides an important basis for identifying the genetic mechanisms of drought tolerance.

Drought stress often causes stomata closure and leaf wilting due to cell dehydration and loss of turgor, negatively influencing photosynthetic capacity (Xu et al., 2010). The perturbation of photosynthesis can be associated with changes to the biochemical reaction of photosystem II, which can be assessed by chlorophyll fluorescence. Alterations in chlorophyll fluorescence parameters have been used to evaluate plant drought tolerance (Maury et al., 1996; Li et al., 2006; O'Neill et al., 2006; Luo et al., 2011; Roostaei et al., 2011). Several QTLs for chlorophyll fluorescence have been identified in different plant species under drought stress (Yang et al., 2007; Kiani et al., 2008; De Miguel et al., 2014; Sukumaran et al., 2016). For example, a large number of QTLs for chlorophyll fluorescence parameters were detected in *Pinus pinaster* under drought stress, which cumulatively explained up to 44% of the observed phenotypic variance (De Miguel et al., 2014). In wheat, 14 additive QTLs and 25 pairs of epistatic QTLs for chlorophyll fluorescence kinetics were identified on under well-watered and drought stress conditions, and explained 8.4–72.7% of the phenotype variation (Yang et al., 2007). These QTLs, located on different chromosomal regions, suggest that genetic control of the expression of chlorophyll fluorescence differed under different water conditions (Yang et al., 2007).

Maintenance of adequate plant water status is critical for plant drought tolerance. Genetic variation in whole-plant water use and cellular water retention allows QTLs to be identified under drought conditions (Viger et al., 2013; De Miguel et al., 2014; Kapanigowda et al., 2014; Merewitz et al., 2014; Iglesias-García et al., 2015). When sorghum was grown under 40 and 80% of field capacity, 3 QTLs associated with the ratio of CO_2_ assimilation to transpiration co-localized with agronomic traits such as leaf area and biomass, and accounted for 17–21% of the phenotypic variation in them (Kapanigowda et al., 2014). Several QTLs for leaf relative water content were also detected in pea (Iglesias-García et al., 2015) and one in barley (Fan et al., 2015). In creeping bentgrass, the detected QTLs were closely associated with drought tolerance traits related to water use and water maintenance, including water use efficiency, canopy temperature depression, and relative water content (Merewitz et al., 2014). The results indicate that genetic control of plant water relations plays an important role in conferring drought tolerance both in annual and perennial grass species.

*Brachypodium distachyon* (Brachypodium) is a temperate, wild grass species. This species has a small genome size, a fully sequenced genome, small stature, a short-life cycle for many genotypes, and a high recombination rate (Draper et al., 2001; Garvin et al., 2008; Vogel et al., 2010; Huo et al., 2011). Since it is phylogenetically closer to many economically important turf, forage, and bioenergy grasses than is rice (Draper et al., 2001), it can be employed as a research surrogate for grass species without genome sequence information. In addition, due to the presence of distinct winter and spring habit genotypes that may differ in adaptation to adverse environments, Brachypodium is an attractive model plant for examining plant responses to abiotic stresses such as drought tolerance. Brachypodium accessions were found to vary significantly in whole-plant responses to drought stress as assessed by leaf water content and chlorophyll fluorescence (Luo et al., 2011). To date, only one published report identified QTLs associated with water use efficiency in Brachypodium under dry environments (Des Marais et al., 2016). In this study, we identified QTLs associated with drought tolerance using a RIL population created from a cross between genotype Bd3-1 (drought-sensitive) and genotype Bd1-1 (drought tolerant) (Luo et al., 2011, 2016). Identifying QTLs for drought tolerance will provide insights into genetic control of drought tolerance in Brachypodium.

## Materials and methods

### Plant materials

A recombinant inbred line (RIL) population of Brachypodium was generated from a cross between inbred genotypes Bd3-1 (female) and Bd1-1 (male), two lines contrasting drought tolerance (Luo et al., 2011, 2016). The population was F_5:6_ generation, and contained 95 RILs. Two experiments were conducted for phenotypic evaluation and QTL identification using this mapping population in a greenhouse at Purdue University, West Lafayette, IN, USA.

Seeds of RILs were sown in tubes (4 cm diameter and 21 cm deep) containing a sandy-loam soil with a pH of 6.9. Each tube had the same volume of soil and one uniformed plant. Seeding was performed on 2 May 2014 for Experiment 1 (Exp 1) and 15 October 2016 for experiment 2 (Exp 2). Plants were watered every 2 days and fertilized once a week with a soluble fertilizer (N- P_2_O_5_-K_2_O, 24-8-16; Scotts Inc., Marysville, OH, USA) and micronutrients at the rate of approximately 0.25 g nitrogen per liter. During the growing and treatment periods, the average temperatures were 23/20°C for Exp 1 and 20/17°C (day/night) for Exp 2, while photosynthetically active radiation was approximately 550 μmol m^−2^ s^−1^ for Exp 1 and 350 μmol m^−2^ s^−1^ for Exp 2, with a 10 h light period of natural and artificial light.

### Drought treatment

Drought stress treatment began on 2 June 2014 and lasted for 7 days for Exp 1, while for Exp 2, drought stress started on 24 November 2016 and lasted for 8 days. Drought stress was imposed by withholding water from the grasses and ended when permanent wilting (the leaves were no longer rehydrated at night and in the morning) occurred to the most of the plants. The control plants were watered during the treatment.

### Phenotypic trait measurements

At the end of drought stress treatment, leaf wilting (WT) was visually rated on a scale of 0 (no observable wilting) to 3 (severely wilted; Luo et al., 2011). Plant height (HT) was measured from the soil surface to the top of the uppermost leaf blade. Leaf tissues were harvested for determining leaf fresh (FW) and dry weight (DW). Leaf water content (LWC) was determined according to the equation: WC = (FW-DW)/FW × 100, where FW is fresh weight and DW is dry weight. Leaf photochemical efficiency was determined by measuring leaf chlorophyll fluorescence (Fv/Fm) using a fluorescence meter (OS-30P, OPTI-Sciences, Hudson, NH, USA). The measurement was performed at night after the plants were dark-adapted for at least an hour. Each tube was measured one time by randomly clamping three to four leaves.

### Experimental design and data analysis

The experiment was a split-plot design for Exp 1 and Exp 2 with three replicates. The main plot was drought treatment and the subplot was RIL. The individual of RILs was arranged randomly within a treatment. Analysis of variance (ANOVA) was calculated using SAS PROC MIX with replication as random effects. Correlation analysis between parameters was performed using the PROC CORR procedure in Statistical Analysis System (version 9.1; SAS Institute, Cary, NC).

### Genotyping and SNP identification

Genomic DNA of the RILs and their parents was extracted using a DNeasy Plant Mini Kit (Qiagen Inc., Valencia, CA, USA) according to the manufacturer's instructions. Genotyping was conducted using genotyping-by-sequencing (GBS) (Elshire et al., 2011) at the Institute for Genomic Diversity at Cornell University. The GBS library was constructed as previously described (Elshire et al., 2011). In brief, DNA samples were digested with enzyme ApeKI and then ligated to adapters with barcodes using T4 ligase (New England Biolabs, Ipswitch, MA). Primers complementary to the adaptor sequences were used for PCR. The PCR product was sequenced on a Genome Analyzer II (Illumina, San Diego, CA). The resulting reads were filtered and aligned to the Brachypodium reference genome (*Brachypodium distachyon* v. 3.1) for SNP calling, conducted by a TASSELGBS pipeline as previously described (Elshire et al., 2011; Glaubitz et al., 2013).

### Genetic map construction and QTL analysis

After filtering GBS data (heterozygote >2 and missing data >5%), 1935 SNPs were obtained for the RIL population. Redundant SNP markers that did not provide additional recombination information were removed before constructing the linkage map. Ultimately, 467 SNPs was used to construct linkage map and for QTL identification, using QTL ICIMapping (Meng et al., 2015). Inclusive composite interval mapping for additive (ICM-ADD) mapping was used to identify QTLs using a LOD threshold of 3.0.

### Candidate gene identification and allelic variation with traits

QTL “hotspots” were defined where at least one QTL explained >5% of the phenotypic variation of a trait, and where multiple trait QTLs were present in the same region of the genome (Viger et al., 2013). Candidate genes within a QTL region were identified using adjacent markers on the genetic and physical maps and searched against Brachypodium genome. The selection of candidate genes for examining allelic variations was based on putative function for drought tolerance, PCR amplification results, and whether a single long exon exits for a gene that can be sequenced directly using DNA. Finally, 5 candidate genes were chosen for gene sequencing and obtained SNPs (Supplementary Table S1), and these genes are known to play a key role in drought tolerance in plant species (Mao et al., 2010; Baldoni et al., 2015; Saha et al., 2015; Singh and Laxmi, 2015; He et al., 2016).

Genomic DNA was extracted from 56 additional accessions of Brachypodium varying in drought tolerance (Luo et al., 2016), and was used as PCR amplification template for synthesis and sequencing of selected genes. Primers were designed based on a single long exon for a gene and introns were excluded for sequencing (Supplementary Table S1). Sequencing and SNP calling procedures were described previously in Bracchypodium (Luo et al., 2016) and in perennial grass species (Yu et al., 2013). SNP markers with minor allele frequency <5% were filtered. Allelic variations of candidate genes and phenotypic differences of individuals carrying different alleles of these genes were compared under drought stress using PROC GLM in the Statistical Analysis System (version 9.1; SAS Institute, Cary, NC). Phenotypic data of Fv/Fm and LWC under drought stress were adopted from our previous study by Luo et al. (2011).

## Results and discussion

### Phenotypic trait variation and correlation

The mean values of all traits significantly decreased under drought stress, compared to their respective controls in both experiments except for HT and DW in Exp 2. Genotype effects were observed for all traits in both experiments. Significant treatment by genotype interactions were also found in all traits except for HT in Exp 1 and DW in Exp 2. Across the population, leaf wilting (WT) ranged from 0 to 2.3 for Exp 1 and 0 to 2.4 for Exp 2 under drought stress (DS), while all lines varied largely in HT, FW, Fv/Fm, and LWC under well-watered (NS) and DS in both experiments (Table 1). Specifically under DS, values of HT, FW, DW, LWC, and Fv/Fm ranged from 9.5 to 15.0 cm, 0.12 to 0.25 g, 0.04 to 0.13 g, 35.0 to 74.7%, and 0.65 to 0.83 for Exp 1, and ranged from 8.0 to 16.0 cm, 0.18 to 0.44 g, 0.06 to 0.19 g, 34.5 to 78.8%, and 0.63 to 0.81 for Exp 2, respectively. Drought stress reduces water availability, causes loss of turgor, and impairs mitosis, leading to reduced cell elongation, limited cell division, and diminished plant growth (Farooq et al., 2009). Drought tolerant perennial ryegrass showed delayed reductions in plant height and leaf width under drought stress, compared to the sensitive accession, while leaf DW did not alter between the well-watered control and deficit irrigation treatment in the tolerant accession but decreased in sensitive accession under drought stress (Jiang et al., 2016). The results suggested a relationship between plant growth and drought tolerance in perennial grass species.

**Table 1:** **Effects of drought stress on plant height (HT), leaf fresh weight (FW), leaf dry weight (LDW), chlorophyll fluorescence (Fv/Fm), and leaf wilting (WT) of ***Brachypodium distachyon*** recombinant inbred line (RIL) population derived from Bd3-1 × Bd1-1 grown under non-drought stress and drought stress conditions in experiment 1 (Exp 1) and experiment 2 (Exp 2)**.

**Trait**	**Exp 1**					**Exp 2**				
	**Non-drought stress**	**Drought stress**	**Treatment (T)**	**Genotype (G)**	**G × T**	**Non-drought stress**	**Drought stress**	**Treatment (T)**	**Genotype (G)**	**G × T**
HT (cm)	13.3 (9.2–16.7)	12.4 (9.5–15.0)	^*^	^***^	NS	12.1 (8.9–15.0)	12.1 (8.0–16.0)	NS	^***^	^*^
FW (g)	0.62 (0.21–1.38)	0.19 (0.12–0.25)	^**^	^***^	^***^	0.69 (0.21–1.0)	0.31 (0.18–0.44)	^*^	^***^	^**^
DW (g)	0.12 (0.04–0.26)	0.08 (0.04–0.13)	^*^	^***^	^*^	0.15 (0.04–0.23)	0.12 (0.06–0.19)	NS	^***^	NS
LWC (%)	81.4 (76.8–84.1)	54.0 (35.0–74.7)	^***^	^***^	^***^	78.5 (72.8–84.5)	58.1 (34.5–78.8)	^**^	^***^	^**^
Fv/Fm	0.82 (0.80–0.83)	0.78 (0.65–0.83)	^**^	^***^	^***^	0.82 (0.80–0.83)	0.77 (0.63–0.81)	^***^	^***^	^***^
WT	–	1.23 (0–2.33)	–	^***^	–	–	1.23 (0–2.4)	–	^*^	–

*, **, *** *Significance at P < 0.05, 0.01, and 0.001, respectively. NS, not significant*.

**Figure 1:**
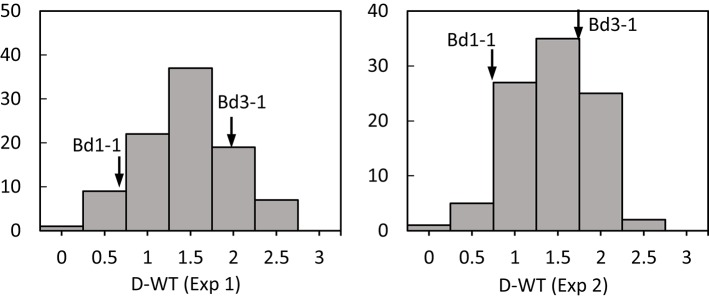
**Distribution of leaf wilting (DT) of ***Brachypodium distachyon*** recombinant inbred line (RIL) population derived from Bd3-1 × Bd1-1 grown under drought stress (D) in experiment 1 (Exp 1) and experiment 2 (Exp 2)**. Arrows indicate Bd1-1 and Bd3-1.

**Figure 2:**
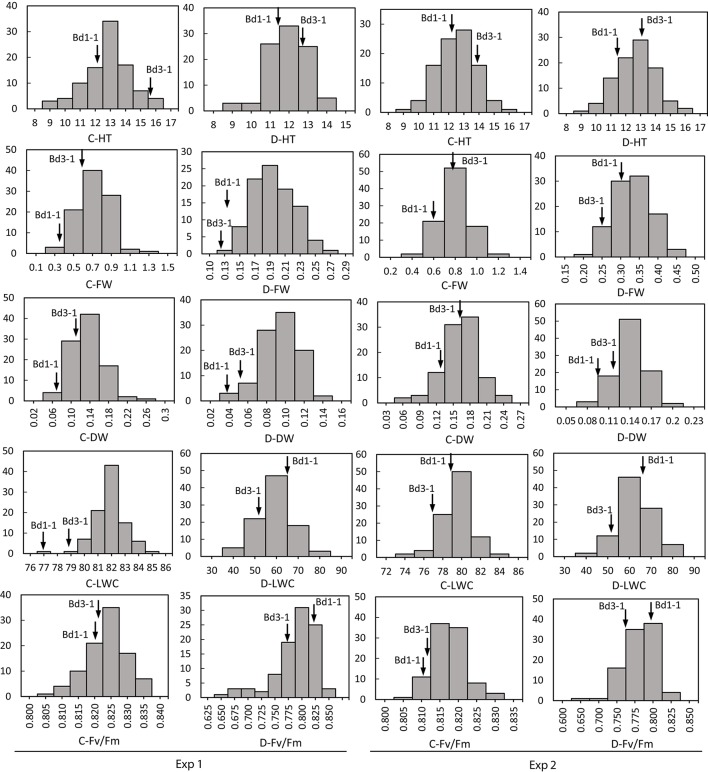
**Distribution of plant height (HT), leaf fresh weight (FW), leaf dry weight (DW), leaf water content (LWC), and chlorophyll fluorescence (Fv/Fm) of ***Brachypodium distachyon*** recombinant inbred line (RIL) population derived from Bd3-1 × Bd1-1 grown under well-watered (C) and drought stress (D) in Experiment 1 (Exp 1) and Experiment 2 (Exp 2)**. Arrows indicate Bd1-1 and Bd3-1.

Transgressive segregation was observed for all traits under normal and stressed environments (Figures 1, 2). All measurements were quantitative traits as shown by normal distributions of their response to both NS and DS. The generally normal distribution of various measurements indicates polygenic segregation for genes controlling growth and physiological traits. The parent Bd 1-1 exhibited less WT than parent Bd3-1, while maintaining higher LWC and Fv/Fm under DS for both experiments (Figures 1, 2). Meanwhile, lower values for HT and DW were found in Bd1-1 than Bd3-1 under NS and DS for both experiments (Figure 2). Reductions in all traits were much more pronounced in Bd3-1 than Bd1-1 under drought stress. Specifically, relative to their controls, percentage reductions of HT, FW, DW, LWC, and Fv/Fm were 20.1, 66.5, 30.3, 14.5, and 0.7% for Bd1-1, and were 30.3, 72.2, 40.6, 28.1, and 6.4% for Bd3-1, respectively. Bd1-1 has a winter annual habit, and Bd3-1 has a spring habit. Our previous research demonstrated that Bd1-1 was more drought tolerant than Bd3-1 by showing less leaf wilting and relatively higher DW, LWC, and Fv/Fm (Luo et al., 2011, 2016). Thus, the results from the present study support the previous observations. A difference in drought tolerance between the two parental lines provides a foundation for a range of drought tolerance traits in segregating population to be characterized and for QTL identification.

Plant height was positively corrected with FW and DW under both NS and DS (Table 1). Leaf wilting is an indicator of plant morphological changes because of cell dehydration and loss of turgor. Negative correlations were found between WT and Fv/Fm (*r* = −0.71) and between WT and LWC (*r* = −0.85), while positive correlation was observed between WT and DW (*r* = 0.71) under DS (Table 2). The results suggest that LWC and Fv/Fm are good parameters for assessing drought tolerance in the RIL population.

**Table 2:** **Pearson correlation coefficients among leaf wilting (WT), plant height (HT), chlorophyll fluorescence (Fv/Fm), leaf fresh weight (FW), leaf dry weight (DW), and leaf water content (LWC) across ***Brachypodium distachyon*** recombinant inbred line (RIL) population derived from Bd3–1 × Bd1-1 under the non-drought control (upper diagonal) and drought condition (bold, lower diagonal)**.

**Trait**	**WT**	**HT**	**Fv/Fm**	**FW**	**DW**	**LWC**
WT		−	−	−	−	−
HT	0.33^**^		0.01	0.62^***^	0.62^***^	−0.16
Fv/Fm	−0.71^***^	−**0.20**		0.05	0.05	−0.0002
FW	−**0.05**	0.40^***^	**0.20**		0.97^***^	−0.16
DW	0.71^***^	0.46^***^	−0.57^***^	0.46^***^		−0.36^***^
WC	−0.85^***^	−0.24^*^	0.84^***^	0.21^*^	−0.73^***^	

*, **, *** *Significance at P < 0.05, 0.01, and 0.001, respectively. NS, not significant*.

*Data averaged experiment 1 and 2*.

### GBS markers and linkage map development

GBS produced 62,737 SNP markers for this RIL population. After filtering markers with heterozygote >2 and missing data >5%, 1,935 SNP markers were used for initial constructing the linkage map. After removing clustered and redundant markers that did not contribute additional recombination information, a total of 467 SNPs was selected for constructing the linkage map and for QTL identification. The Bd3-1/Bd1-1 map spanned 1,618 cM and had an average distance of 3.5 cM between adjacent SNPs (Table 3, Supplementary Figure S1). Chromosome 5 was the shortest in length at 162.4 cM, while chromosome 1 was the longest at 442.4 cM (Table 3). The average distance between markers ranged from 2.79 cM on chromosome 4 to 4.51 cM on chromosome 5. The number of markers were 113, 107, 102, 109, and 36 for chromosomes 1, 2, 3, 4, and 5, respectively. The physical distance was 74.0, 58.6, 58.2, 48.5, and 26.6 Mb and distance (Kb) per marker was 654.9, 547.7, 570.6, 445.0, and 738.9 for chromosomes 1, 2, 3, 4, and 5, respectively (Table 3). The recombination rate was similar around 6.0–6.3 across five chromosomes (Table 3). A plot of genetic distance vs. physical distance suggested that SNPs had good coverage for chromosomes 1–4 (Supplementary Figure S2). It appeared that the centromere region had lower recombination rates than the chromosome arms, as indicated by the slower increase of genetic distance over this part of the chromosome (Supplementary Figure S2).

**Table 3:** **Linkage groups of the developed linkage map and marker distribution in 95 ***Brachypodium distachyon*** recombinant inbred line (RIL) population derived from Bd3-1 × Bd1-1 genotyped by genotyping by sequencing**.

**Linkage groups**	**Number of markers**	**Length (cM)**	**cM/marker**	**Physical size (Mb)**	**Kb/marker**	**Recombination rate (cM/Mb)**
Bd1	113	442.4	3.92	74.0	654.9	6.0
Bd2	107	354.5	3.31	58.6	547.7	6.0
Bd3	102	354.4	3.47	58.2	570.6	6.1
Bd4	109	304.4	2.79	48.5	445.0	6.3
Bd5	36	162.4	4.51	26.6	738.9	6.1

Compared our linkage map to the map of Bd3-1 × Bd21 F_6:7_ RIL population created by 570 SNP markers in Brachypodium (Cui et al., 2012), chromosome 4 had very a similar genetic distance (304.4 vs. 304.7 cM) between the two maps, while the differences in genetic distance were between 33.5 and 66.1 cM for other chromosomes. The physical distance and the recombination rate were similar between the two maps, despite that the two mapping populations differed in one parent. The two maps also had similar patterns of recombination rate except for chromosome 5, whereas more markers were observed in Bd3-1 × Bd21 map (Supplementary Figure S2).

### QTLs for phenotypic traits

Using the Bd3-1 × Bd1-1 RIL population, 26 QTLs were detected on chromosome 1, 2, and 3 in two experiments, with 14 QTLs under NS and 12 QTLs under DS (Table 4). Two QTLs of *C-HT2.1* and *C-HT2.2* were detected on chromosome 2 under NS in Exp 1 with a peak position at 343 and 344 cM, while one QTL of *D-HT2.3* was identified under DS condition in Exp 2 with a peak position at 76 cM (Table 4). LOD scores of these QTLs ranged from 3.6 to 3.7, accounting for 13–16% of the phenotypic variation (PVE).

**Table 4:** **Location and description of quantitative trait loci (QTLs) in ***Brachypodium distachyon*** recombinant inbred line (RIL) population derived from Bd1-1 × Bd3-1 grown under well-watered control (C) and drought stress (D) conditions in experiment I (Exp 1) and experiment 2 (Exp 2)**.

**Trait**	**Trait**	**Exp**	**Chr**.	**Position (cM)**	**Confidence interval (cM)**	**Interval length (cM)**	**QTL start (bp)**	**QTL end (bp)**	**LOD**	**PVE (%)**	**Additive effect**
HT	*C-HT2.1*	1	2	344	342.4–344.0	1.6	B2_57293448	B2_57420530	3.58	12.5	−0.52
	*D-HT2.2*	1	2	343	342.4–344.0	1.6	B2_57293448	B2_57420530	3.68	16.5	−0.43
	*D-HT2.3*	2	2	76	75.7–77.4	1.7	B2_7155713	B2_7437034	3.55	16.0	−0.57
	*C-HT3.1*	1	3	95	94.7–95.8	1.1	B3_11270923	B3_11746359	4.87	18.1	−0.63
FW	*C-FW1.1*	1	1	35	32.9–40.6	7.7	B1_3431431	B1_4132932	3.17	9.17	−0.06
	*C-FW1.2*	1	1	214	213.4–215.1	1.7	B1_33900953	B1_34018497	3.14	9.08	0.06
	*D-FW1.3*	2	1	101	101–105.3	4.3	B1_12247879	B1_13815258	3.01	13.6	0.02
	*C-FW2.1*	1	2	189	188.3–190.1	1.8	B2_31815569	B2_33332731	7.85	24.7	−0.09
	*C-FW2.2*	2	2	185	183.8–188.3	4.5	B2_30817239	B2_31815569	3.96	19.1	−0.06
	*C-FW3.1*	1	3	90	76.3–91.8	15.5	B3_6581022	B3_9345741	6.26	19.7	−0.08
DW	*C-DW1.1*	1	1	38	32.9–40.6	7.7	B1_3431431	B1_4132932	4.89	17.4	−0.02
	*C-DW1.2*	1	2	189	188.3–190.1	1.8	B2_31815569	B2_33332731	7.52	27.3	−0.02
	*C-DW2.1*	2	2	185	183.8–188.3	4.5	B2_30817239	B2_31815569	3.74	18.2	−0.02
	*C-DW3.1*	1	3	72	70.7–76.3	5.6	B3_6022471	B3_6581022	3.01	9.67	−0.01
	*D-DW3.2*	1	3	92	91.8–93.5	1.7	B3_9345741	B3_11215196	4.42	19.8	−0.01
LWC	*C-LWC2.1*	1	2	38	31.2–41.1	9.9	B2_3296983	B2_4223689	3.77	16.1	0.46
	*D-LWC2.2*	1	2	182	177.7–182.7	5.0	B2_25605715	B2_30714160	3.16	11.0	2.78
	*D-LWC3.1*	1	3	91	76.3–91.8	15.5	B3_6581022	B3_9345741	4.36	16.0	3.34
	*D-LWC3.2*	2	3	178	160–179.7	19.7	B3_32337330	B3_36933812	3.55	13.6	2.99
Fv/Fm	*D-Fv/Fm2.1*	1	2	182	177.7–182.7	5.0	B2_25605715	B2_30714160	4.09	18.7	0.02
	*C-Fv/Fm2.2*	2	2	304	302.1–304.4	2.3	B2_53290301	B2_53567342	3.36	13.4	−0.002
	*C-Fv/Fm3.1*	1	3	123	122.8–125.7	2.9	B3_16046401	B3_16869416	3.39	15.3	−0.003
	*D-Fv/Fm3.2*	2	3	112	111.6–119.3	7.7	B3_13878583	B3_14553034	3.79	17.2	0.12
WT	*D-WT2.1*	1	2	182	177.7–182.7	5.0	B2_25605715	B2_30714160	3.56	16.6	−0.22
	*D-WT2.2*	2	2	199	198.6–199.2	0.6	B2_34592178	B2_34588824	3.25	11.4	−0.15
	*D-WT3.1*	2	3	181	179.7–182.7	3.0	B3_36933812	B3_37856612	3.12	11.9	−0.15

*PVE, percentage of phenotypic variance explained by each QTL*.

*LOD, logarithm of odds*.

*HT, plant height; FW, leaf fresh weight; DW, leaf dry weight; LWC, leaf water content; Fv/Fm, chlorophyll fluorescence; WT, leaf wilting*.

Six QTLs for FW were detected on chromosomes 1, 2, and 3 with LOD scores >3.0 and PVE-values from 9.1 to 19.7%, including *C-FW1.1, C-FW1.2, C-FW2.1, C-FW3.1, C-FW2.2* under NS, and *D-FW1.3* under DS (Table 4). For DW, *C-DW1.1, C-DW1.2, C-DW2.1*, and *C-FW3.1* under NS and *D-DW3.2* under DS were located on chromosomes 1, 2, and 3, with LOD scores ranging from 3.0 to 4.9 and PVE-values from 9.7 to 27.3% in Exp 1 or Exp 2 (Table 4). Among QTLs for FW and DW, *C-FW2.1* and *C-DW1.2* were co-localized on chromosome 2 with a peak position at 189 cM for Exp 1, while *C-FW2.2* and *C-DW2.1* were co-localized on chromosome 2 with a peak position at 185 cM for Exp 2. Combined, these QTLs accounted for 25–27% (Exp 1) and 18–19% (Exp 2) of phenotypic variation.

Three QTLs named *C-LWC2.1, D-LWC2.2*, and *D-LWC3.1* were located on chromosomes 2 and 3 for Exp 1 and one QTL named *D-LWC3.2* on chromosomes 3 for Exp 2 (Table 4). These QTLs explained between 11.0 and 16.1% of the phenotypic variation. Particularly, *D-LWC2.2* peaked at 182 cM was near to *C-FW2.1* and *C-DW1.2* at 189 cM. In Brachypodium, Des Marais et al. (2016) identified four QTLs for Δ^13^C, an indicator of water use efficiency, on chromosome 2, 3, and 5 in a RIL population derived from Bd3-1 × Bd21, which explained 9.3–19.4% of the phenotypic variation. The RIL population used in the study mentioned above was developed from the parents that are both drought sensitive (Luo et al., 2011). We did not detect any QTLs on chromosome 4 and 5 in this study.

For Fv/Fm under NS, *C-Fv/Fm3.1* was detected on chromosome 3 for Exp 1 and C-*Fv/Fm2.2* on chromosome 2 for Exp 2. Under DS, *D-Fv/Fm2.1* was mapped to chromosome 2 at 182 cM with LOD score of 4.1 and PVE-value of 18.7% for Exp 1, while *D-Fv/Fm3.2* was detected on chromosome 3 at 112 cM with LOD score of 3.8 and PVE-value of 17.2% for Exp 2. Three QTLs was associated with WT. One *D-WT2.1* was located on chromosome 2 at 182 cM for Exp 1 and explained 16.6% of WT. For Exp 2, *D-WT2.2* was located on chromosome 2 at 199 cM and explained 11.4% of WT, while *D-WT3.1* was located on chromosome 3 at cM 181, explained 11.9% of WT.

**Figure 3:**
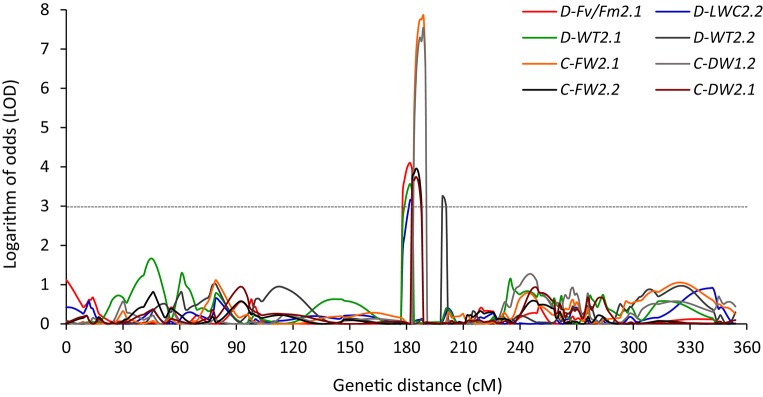
**QTLs detected between 182 to 199 cM on chromosome 2 for leaf wilting (WT), chlorophyll fluorescence (Fv/Fm), leaf water content (LWC), leaf fresh weight (LFW), and leaf dry weight (LDW) under well-watered (C) and drought stress (D) for Experiment 1 (***D-Fv/Fm2.1***, ***D-LWC2.2***, ***D-WT2.1***, ***C-FW2.1***, and ***C-DW1.2***) and Experiment 2 (***D-WT2.2***, ***C-FW2.2***, and ***C-DW2.1***) in ***Brachypodium distachyon*** recombinant inbred line (RIL) population derived from Bd3-1 × Bd1-1**.

Several cases of clustering of QTLs were found under NS and/or DS in both experiments. Within a region of 12.4 cM (177.7–190.1 cM), a QTL located on chromosome 2 for C-FW, C-DW, D-LWC, D-Fv/Fm, and D-WT was found in both experiments (Table 4, Figure 3). In Exp 1, 3 QTLs of *D-WT2.1, D-LWC2.2*, and *D-Fv/Fm2.1* co-localized on chromosome 2 at peak of 182 cM, spanning the approximate regions of 5 cM (Table 4; Figure 3). Since WT, Fv/Fm and LWC are associated with drought tolerance of Brachypodium (Luo et al., 2011, 2016), the results suggest that there may be genes that simultaneously influence these traits. Similarly, in a region of 12.7 cM, a QTL for D-LWC and WT was identified on chromosome 3 in both experiments. Together with *D-WT2.1* on chromosome 2, the results indicate that visual rating is appropriate for QTL identification for drought tolerance. The same QTLs for visual plant wilting and for relative water content under drought stress was also identified in barley and accounted for 14–45% of phenotypic variation (Fan et al., 2015), supporting the observation in this study.

Co-localization of QTLs for physiological traits and/or with agronomic traits have been reported in plants exposed to drought stress, suggesting a link between individual QTLs and multiple traits (Viger et al., 2013; Borrell et al., 2014; Kapanigowda et al., 2014; Merewitz et al., 2014; Khan et al., 2015). Overlapping QTLs for the chlorophyll fluorescence parameters and plant water status traits were identified in sunflower under drought stress (Kiani et al., 2008). QTLs for transpiration ratio were also associated with leaf area and biomass (Kapanigowda et al., 2014) and QTLs for chlorophyll content co-localized with flowering time in sorghum (Mace et al., 2012; Sukumaran et al., 2016). QTLs for relative water content, normalized difference vegetation index, and chlorophyll content were co-localized in creeping bentgrass (Merewitz et al., 2014). In our study, in addition to co-localization of QTLs, most of the QTLs were specific for one water treatment condition (well-watered or drought), demonstrating that QTLs exhibited different patterns for genetic control of physiological traits such as LWC and Fv/Fm under variable water regimes.

### Candidate genes and allelic variation with drought tolerance

Since *D-LWC2.2, D-Fv/Fm2.1*, and *D-WT2.1* were all located on chromosome 2 at peak position 182 cM in Exp 1, searching for candidate genes for drought tolerance was conducted in the region spanning 177.7–182.7 cM. A total of 164 annotated genes were found in this region, including WRKY, MYB, NAC, MADS, ethylene-responsive transcription factors (ERF), dehydration-responsive element-binding protein 2B-like (DREB2B), late embryogenesis abundant protein (LEA), protein dehydration-induced 19-related (DI19), calcium/calmodulin-dependent protein kinase (CAMK), receptor-like protein kinase (RPK), receptor-like serine/threonine protein kinases (SPK), cysteine-rich TM module stress tolerance (CYSTM), histone H2B, and antioxidant enzymes such as glutathione s-transferase (GST) and iron/manganese superoxide dismutases (Fe/Mn SOD). These candidate genes are known to play a key role in drought tolerance in many plant species (Mao et al., 2010, 2015; Licausi et al., 2013; Yu et al., 2013; Baldoni et al., 2015; Singh and Laxmi, 2015; Xu et al., 2015; He et al., 2016; Hettenhausen et al., 2016; Lu et al., 2016).

**Figure 4:**
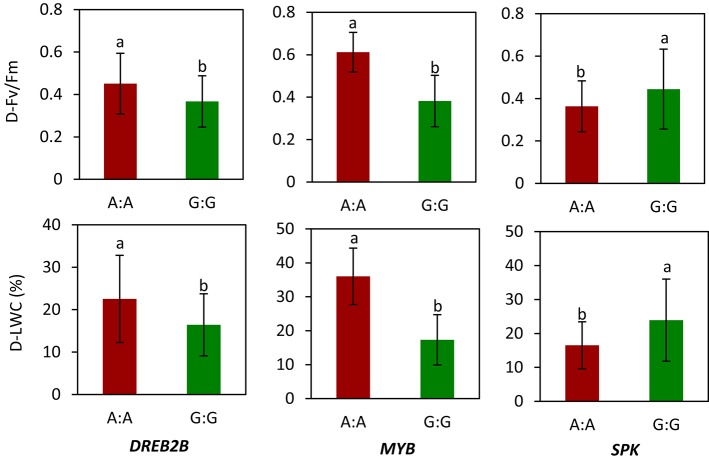
**Allelic variation of candidate genes in relation to values of chlorophyll fluorescence (Fv/Fm) and leaf water content (LWC) in ***Brachypodium distachyon*** natural accessions under drought stress (D)**. MYB transcription factor (*MYB*); dehydration-responsive element-binding protein 2B-like (*DREB2B*); receptor-like serine threonine kinases (*RLK*). Columns with the same letter were not significantly different at *P* < 0.05. Bars indicate standard deviation. Data of Fv/Fm and LWC under drought stress were adopted from our previous study by Luo et al. (2011).

We selected *WRKY, MYB, MADS, DREB2B*, and *SPK* for gene sequencing and obtained SNPs. The selection of these genes was described previously. Of these genes, allelic variations in *DREB2B, MYB*, and *SPK* showed a relationship with LWC and Fv/Fm in 56 accessions under drought stress. Specifically, genotype carrying A:A in *DREB2B* had a higher mean of D-Fv/Fm (0.45) and D-LWC (22.5%) than genotypes carrying G:G (0.37 for D-Fv/Fm and 16.4% for D-LWC) under drought stress (Figure 4). For *MYB*, the mean D-Fv/Fm and D-LWC were 0.61 and 36.0% for genotype A:A and 0.38 and 17.3% for genotype G:G, respectively. For *SPK*, genotype G:G had a mean D-Fv/Fm of 0.44 and D-LWC of 23.9%, while genotype A:A had a mean Fv/Fm of 0.36 and D-LWC of 16.5% under drought stress.

Transcription factors (TFs) generally act as key regulators of gene expression. Dehydration-responsive element binding protein (DREB)/CBF (C-repeat binding factor) regulons function in abscisic acid (ABA)-independent regulation of gene expression under drought stress (Nakashima et al., 2009). Overexpression of *DREB2* induced up-regulation of stress-inducible genes and improved drought tolerance of *Arabidopsis* and soybean (Sakuma et al., 2006; Engels et al., 2013). At population level, natural variations in the promoter region of *ZmDREB2.7* contributed to drought tolerance in maize (Liu et al., 2013). The majority of the MYB proteins in the plants belong to the R2R3-MYB subfamily (Baldoni et al., 2015; Roy, 2016). In *Arabidopsis*, AtMYB96 regulated lateral root meristem activation under drought stress through an ABA-auxin signaling crosstalk pathway (Seo et al., 2009). Overexpression of a *MYB* conferred drought tolerance by increasing sugar, proline, and abscisic acid contents, decreasing lipid peroxidation, and regulating expression of ABA biosynthesis genes and other signaling and drought responsive genes (Zhang et al., 2012; Sun et al., 2014; Xiong et al., 2014; Baldoni et al., 2015). The SnRK2 family members are plant-specific serine/threonine kinases involved in plant response to abiotic stresses and ABA-dependent plant development (Kulik et al., 2011). SnRK2 and SnRK3 play a role in plant responses to environmental stresses (Hrabak et al., 2003). The transgenic *Arabidopsis* carrying *TaSnRK2.4* decreased rate of water loss, maintained higher cell membrane stability and photosynthesis potential, and increased osmotic potential (Mao et al., 2010). In this study, the higher values of Fv/Fm and LWC in some genotypes under drought stress than the other genotypes demonstrated that allelic variations of these genes could contribute to natural variation of physiological traits associated with drought tolerance in Brachypodium. Collectively, the results suggest a positive role of these genes in improving general fitness of the plants under water deficit conditions.

## Conclusions

A linkage map of the RIL Brachypodium population Bd3-1 × Bd1-1 spanning 1,618 cM was constructed using 467 SNP markers. This genetic map was used to identify QTLs for traits of interest associated with drought tolerance. Twenty-six QTLs were detected on chromosomes 1, 2 and 3, with 14 QTLs under well-watered condition and 12 QTLs under drought stress. QTLs for WT, LWC, and Fv/Fm under drought stress were associated with drought tolerance. Allelic diversity of *DREM2B, MYB*, and *SPK* may play a role in explaining natural variation of whole plant drought tolerance in Brachypodium. The QTLs detected in this study provide an important first step in identifying the molecular basis of drought tolerance and further elucidating genetic control of drought tolerance in this model grass species.

## Author contributions

YJ designed the experiments and led writing of the manuscript; XW and XZ collected phenotypic data; XW, XZ, XY, NL, ZP, and HL analyzed sequence data and performed QTL analysis; DG developed the mapping population and participated in interpreting results and writing the manuscript. All authors approved the manuscript.

### Conflict of interest statement

The authors declare that the research was conducted in the absence of any commercial or financial relationships that could be construed as a potential conflict of interest.
