# Supplementary material for: Quantitative Trait Loci Associated with Drought Tolerance in Brachypodium distachyon
Source: Front Plant Sci. 2017 May 17;8:811. doi: 10.3389/fpls.2017.00811 (PMC5434166; doi:10.3389/fpls.2017.00811)
Supplement: Supplementary file 1 [file Table1.pdf]

Table S1. Primers used for amplification of candidate genes and sequencing results

Gene	Species	Genbank	Primer	Size (bp)	Sequencing length (bp)	No. of SNP
WRKY	Brachypodium	XM_003566336.3	F: ACGTCCTTGCACCATACG<br>R: TGCAACACATCGGCTCCA	427	392	3
WRKY	Brachypodium	XM_003566336.3	F: ACAAGAACTACTACGGGTGC<br>R:GATGAAAAGGGAAGATGCCG	462	435	4
MYB	Brachypodium	XM_003566307.3	F: GGAACTGGAGCGTGATCAG<br>R: CACTGCTCGATCGATCCTG	728	699	4
SPK	Brachypodium	XM_010233179.1	F: TCGTCCTCTGTTCAAAGACC<br>R: TCAGAGGTCCTTGAAGCTTC	483	454	1
DREB2B	Brachypodium	XM_003568607.3	F:CAAAGGGTTCAAAGAAGGGC<br>R: AGGTATGAAGGCTCCTGTTG	676	650	2
MADS	Brachypodium	XM_001301399.1	F: AGCGGATAGAGAACAAGGA<br>R: GCCCTGTTCTGTCCTACTTT	491	461	5
