# Supplementary material for: Quantitative Trait Loci Associated with Drought Tolerance in Brachypodium distachyon
Source: Front Plant Sci. 2017 May 17;8:811. doi: 10.3389/fpls.2017.00811 (PMC5434166; doi:10.3389/fpls.2017.00811)
Supplement: Supplementary file 2 [file Image1.pdf]

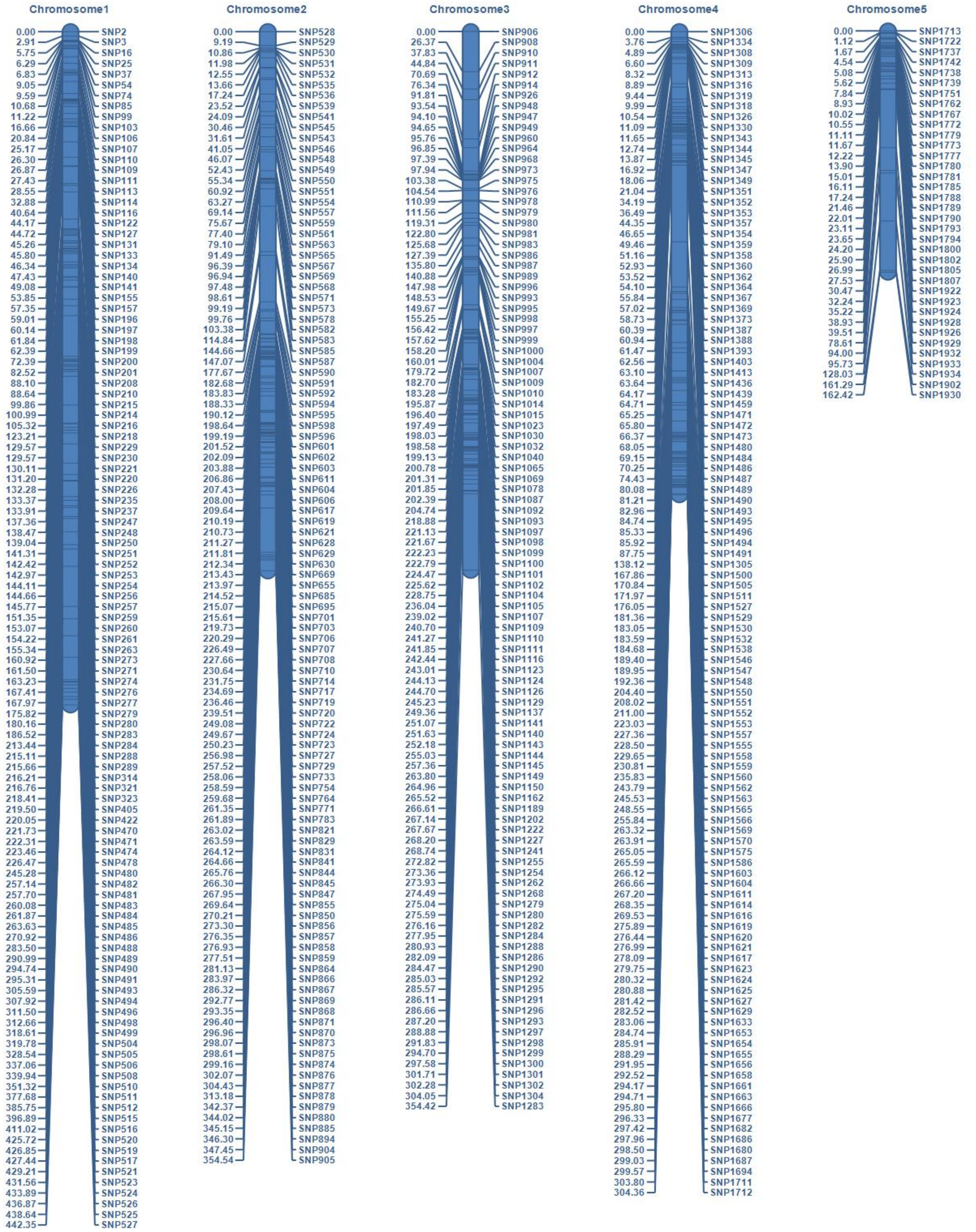

Supplemental Fig. S1. A linkage map of *Brachypodium distachyon* RIL population developed from Bd3-1 × Bd1-1. The map was constructed using 467 single SNP markers obtained from genotyping by sequencing.

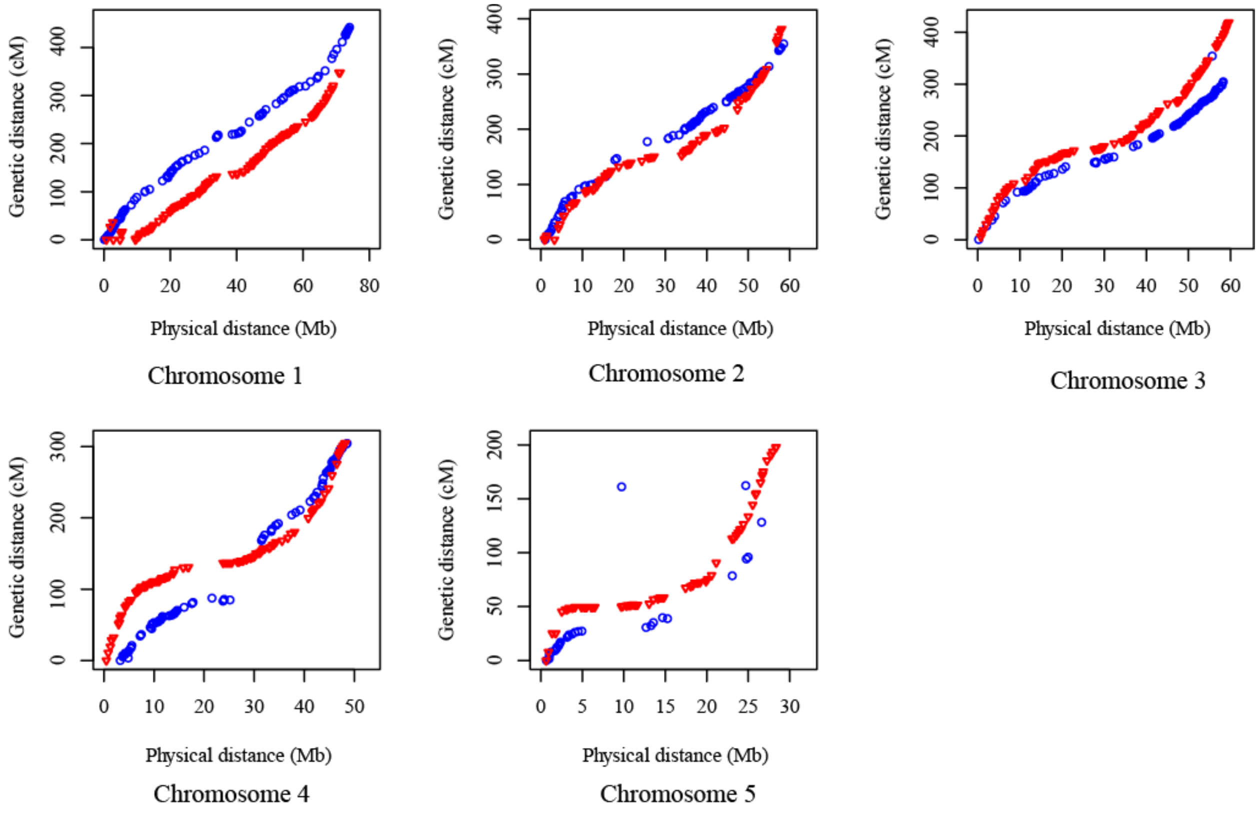

Supplemental Fig. S2. Comparison of genetic and physical distance of the 5 Brachypodium chromosomes of linkage maps of Bd1-1 × Bd3-1 (blue color, this study) RIL using 467 SNP markers and Bd3-1 × Bd21 (red color) RIL using 570 SNP markers from Cui et al. (2012).
